# Supplementary material for: The Effect of Conductive Additive Morphology and Crystallinity on the Electrochemical Performance of Ni-Rich Cathodes for Sulfide All-Solid-State Lithium-Ion Batteries
Source: Nanomaterials (Basel). 2023 Dec 1;13(23):3065. doi: 10.3390/nano13233065 (PMC10708284; doi:10.3390/nano13233065)
Supplement: Supplementary file 1 [file nanomaterials-13-03065-s001.zip › nanomaterials-2742384-supplementary.pdf]

Supplementary Data

The Effect of the Conductive Additive Morphology and Crystallinity on the Electrochemical Performance of Ni-rich Cathodes for Sulfide All-Solid-State Lithium-ion Batteries

Jae Hong Choi [1], Sumyeong Choi [1], Tom James Embleton [1], Kyungmok Ko [1], Kashif Saleem Saqib [1], Jahanzaib Ali [1], Mina Jo [1], Junhyeok Hwang [1], Sungwoo Park [1], Minhu Kim [1], Mingi Hwang [1], Heesoo Lim [1] and and Pilgun Oh [*1,2]

[1]Department of Smart Green Technology Engineering, [2]Department of Nanotechnology Engineering, Pukyong National University, 45, Yongso-ro, Nam-gu, Busan 48547, Republic of Korea

[3] Department of Electrical Engineering, Chosun University, 309, Pilmun-daero, Dong-gu, Gwangju 61452, Republic of Korea

*Correspondence: Tel.:+82-51-629-6387; Fax: +82-51-629-6388 *E-mail addresses*: poh@pknu.ac.kr

Figure S1.

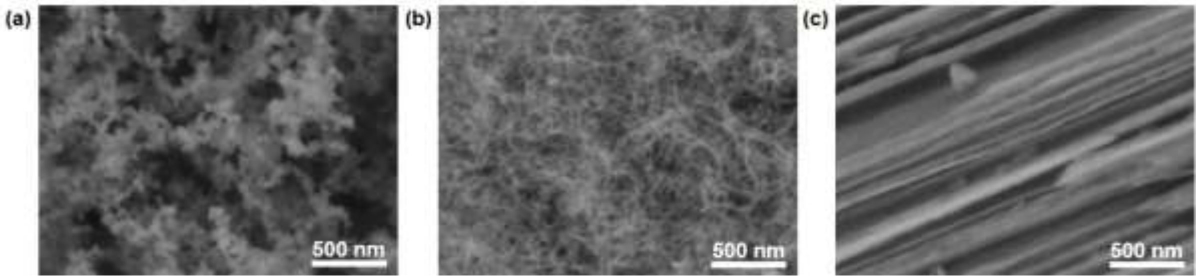

Figure S1 - High magnification scanning electron microscopy (SEM) imaging of the carbon surface of a) Super C, b) CNTs, and c) CNF

Table S1.

Table S1 – Comparison between this work and related publications applying sulfide solid electrolyte and $LiNi_{0.8}Co_{0.1}Mn_{0.1}O_2$ in all-solid-state lithium-ion battery systems

Reference	Cathode Active Material (AM)	Solid Electrolyte (SE)	Conductive Additive (CA)	Mass Ratio (AM:SE:CA:BM)	Discharge Capacity (mAh g^{-1})	ICE	C-Rate	Volt. Range (vs. Li/Li^+)	Retention	C-Rate	Temperature
S1 [1]	NCM811	$Li_{10}SnP_2S_{12}$	AB	50:48:2:0	165	67.9%	0.1C	2.85-4.35V	77.8%@100cyc	0.1C	30°C
S2 [2]	NCM811	Li_6PS_5Cl	Super P	70:29:1	175	84.1%	0.1C	2.52-4.22V	--	0.1C	25°C
S3 [3]	NCM811	Li_6PS_5Cl	VGCF	70:30:0:0	162.3	76.8%	0.1C	2.62-4.22V	71.8%@270cyc	1C	30°C
S4 [4]	NCM811	$75Li_2S$-$25P_2S_5$	Denka black	60:35:5:0	141	61.3%	0.1C	2.52-4.22V	93.75%@40cyc	0.1C	RT
S5 [5]	NCM811	$75Li_2S$-$25P_2S_5$	Denka black	70:30:0:0	124	70.5%	0.1C	2.7-4.3V	65.3%@50cyc	0.1C	25°C
S6 [6]	NCM811	Li_6PS_5Cl	Super P	70:28:02	157	66.2%	0.1C	2.62-4.42V	63%@30cyc	0.1C	RT
S7 [7]	NCM811	Li_6PS_5Cl<br>$Li_{5.6}PS_{4.6}S_5Cl_{1.0}Br_{0.4}$	VGNF carbon	70:27:3:0	181.2<br>188.4	67%<br>64%	0.1C	2.62-4.4V	75.3%@50cyc<br>80.6%@50cyc	0.1C	25°C
This work	NCM811	Li_6PS_5Cl	CNF<br>CB<br>CNT	70:30:5:0	163.6<br>111.8<br>137.8	85.98%<br>70.45%<br>77.58%	0.05	2.5-4.3V<br>2.5-4.3V<br>2.5-4.3V	83.9%@50cyc<br>72.6%@50cyc<br>67.4%@50cyc	0.5C	30°C

Figure S2.

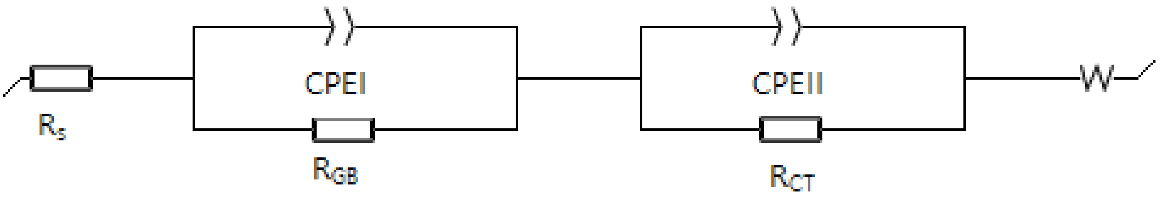

Figure S2. Equivalent circuit diagram associated with the electrochemical impedance spectroscopy (EIS) analysis conducted and provided in Figure 3 of the main manuscript.

Table S2.

Table S2 – The charge/discharge capacity data of the cycles outlined in Figure 5 of the main manuscript

Samples	1st Cycle		10th Cycle		20th Cycle		30th Cycle		40th Cycle		50th Cycle	
	Charge Capacity	Discharge Capacity	Charge Capacity	Discharge Capacity	Charge Capacity	Discharge Capacity	Charge Capacity	Discharge Capacity	Charge Capacity	Discharge Capacity	Charge Capacity	Discharge Capacity
NCM-CNF	188.2	163.6	157.8	157.4	151.8	151.6	146.7	146.4	141.9	141.5	137.5	137.3
NCM-CNTs	173.8	137.7	124.8	123.6	113.4	112.5	105.1	104.4	98.5	98.0	93.4	92.9
NCM-CB	141.1	111.81	100.9	99.6	94.4	92.8	88.9	87.5	85.3	84.2	81.6	81.2

References

1. X. Liu, B. Zheng, J. Zhao, W. Zhao, Z. Liang, Y. Su, C. Xie, K. Zhou, Y. Xiang, J. Zhu, H. Wang, G. Zhong, Z. Gong, J. Huang and Y. Yang, "Electrochemo-Mechanical Effects on Structural Integrity of Ni-Rich Cathodes with Different Microstructures in All Solid-State Batteries," *Advanced Energy Materials*, vol. 11, no. 8, pp. 2003583, 2021.
2. Y. S. Park, J. M. Lee, E. J. Yi, J.-W. Moon and H. Hwang, "All-Solid-State Lithium-Ion Batteries with Oxide/Sulfide Composite Electrolytes," *Materials*, vol. 14, no. 8, pp. 1998, 2021.
3. X. Liu, J. Shi, B. Zheng, Z. Chen, Y. Su, M. Zhang, C. Xie, M. Su and Y. Yang, "Constructing a High-Energy and Durable Single-Crystal NCM811 Cathode for All-Solid-State Batteries by a Surface Engineering Strategy," *ACS Applied Materials & Interfaces*, vol. 13, no. 35, pp. 41669-41679, 2021.
4. S. Deng, X. Li, Z. Ren, W. Li, J. Luo, J. Liang, J. Liang, M. N. Banis, M. Li, Y. Zhao, X. Li, C. Wang, Y. Sun, Q. Sun, R. Li, Y. Hu, H. Huang, L. Zhang, S. Lu, J. Luo and X. Sun, "Dual-functional interfaces for highly stable Ni-rich layered cathodes in sulfide all-solid-state batteries," *Energy Storage Materials*, vol. 27, pp. 117-123, 2020.
5. K. Lee, S. Kim, J. Park, S. H. Park, A. Coskun, D. S. Jung, W. Cho and J. W. Choi, "Selection of Binder and Solvent for Solution-Processed All-Solid-State Battery," *Journal of The Electrochemical Society*, vol. 164, no. 9, pp. A2075, 2017.
6. R. Koerver, I. Aygün, T. Leichtweiß, C. Dietrich, W. Zhang, J. O. Binder, P. Hartmann, W. G. Zeier and J. Janek, "Capacity Fade in Solid-State Batteries: Interphase Formation and Chemomechanical Processes in Nickel-Rich Layered Oxide Cathodes and Lithium Thiophosphate Solid Electrolytes," *Chemistry of Materials*, vol. 29, no. 13, pp. 5574-5582, 2017.
7. M.-h. Park, R. Rajagopal and K.-S. Ryu, "Electrochemical performance of the mixed solid electrolyte (100-x)Li3SI-xLi6PS5Cl (x = 0, 10, 20, and 30) for all-solid-state lithium batteries," *Journal of Power Sources*, vol. 501, pp. 230031, 2021.
